# Systematic Review of Chinese Herbal Medicines for Preventing in-Stent Coronary Restenosis after Percutaneous Coronary Intervention

**DOI:** 10.1155/2012/253409

**Published:** 2012-02-16

**Authors:** Guo-Hua Zheng, Jian-Ping Liu, Nissi S. Wang, Hai-Ying Chen, Jian-Feng Chu

**Affiliations:** ^1^The Centre of Evidence Based Medicine, Academy of Integrative Medicine, Fujian University of Traditional Chinese Medicine, NO. 1 Huatuo Road, Shangjie University Town, Fuzhou 350108, China; ^2^The Centre for Evidence-Based Chinese Medicine, Beijing University of Chinese Medicine, Beijing 100029, China; ^3^Pamir Communications, Daly City, CA 94015, USA

## Abstract

Percutaneous coronary intervention (PCI) with stent placement is a standard treatment for coronary artery disease (CAD). In-stent restenosis after PCI remains a challenging clinical problem. In China, Chinese herbal medicines (CHMs) are widely used for preventing restenosis. This paper systematically reviewed the literature on the effectiveness and safety of CHMs in preventing restenosis after PCI in patients with CAD. Electronic databases were searched for randomized controlled trials that compared CHMs plus RWM with the same RWM plus placebo in preventing restenosis after PCI. A total of 52 trials (4905 patients) on 34 CHMs met the inclusion criteria and were analyzed. Ten trials had low risk of bias. Methodological quality of included trials was generally poor. Meta-analysis showed that at the end of at least 3 months' followup, CHMs plus RWM could significantly reduce restenosis rate, cardiac mortality, recurrence rate of angina, acute myocardial infarction, numbers of repeat PCI, and numbers of coronary artery bypass graft. Reported adverse events included gastrointestinal upset, granulocytopenia, and increased alanine transaminase (ALT). CHMs may help prevent restenosis, thus reducing cardiac mortality after PCI. Caution should be exercised in drawing a definitive conclusion due to the poor methodological quality of the trials reviewed.

## 1. Introduction

Coronary artery disease (CAD) is the single leading cause of death and disability in the world [1–3]. Percutaneous coronary intervention (PCI) with stent placement is the standard nonsurgical treatment for CAD, effective in relieving the symptoms of coronary ischemia [4]. But the main limitation of coronary stenting, in particular with bare-metal stents, is in-stent restenosis (ISR) [5]. This is the formation of scar tissue over the stent, which can cause the opened artery to narrow again. The risks of ISR include symptoms of coronary ischemia, often warranting repeat revascularization [6]. With the development and the universal application of the drug-eluting stent (DES), restenosis has been reduced from 10% to 50% for bare-metal stents [7] to <10% for DESs [8]. Despite this improvement, a major drawback of DESs has come to light involving late (after 30 days) and very late (after 1 year) stent thrombosis [9, 10]. In addition, studies have shown that DESs do not reduce late cardiac-related death and the incidence of myocardial infarction (MI) [11–14]. Antithrombotic therapy after PCI, consisting of lifelong aspirin and clopidogrel, is effective in reducing cardiac-related death, MI, and stroke [15]. But dual antiplatelet therapy also has limitations, as stopping prematurely significantly increases the risk of stent thrombosis, MI, and death [16]. Thus, treatment of ISR remains a challenging clinical issue.

In China, Chinese herbal medicines CHMs have a long history of integration with routine Western medical (conventional) interventions. Advancements in such interventions have spurred concomitant application of CHMs in attempts to enhance outcomes. In the past decade, CHMs have been tested in clinical trials as an adjunct therapy for preventing ISR after PCI. CHMs appear to ameliorate ISR after PCI when used alone or combined with routine western medicine (RWM) [16, 17]. Pharmacologic studies have found that some (CHMs) can be administered to dilate coronary vessels, improve circulation, and remove blood stasis (Chinese medicine concept of blood circulation disturbance, resulting in slowing of blood flow, thrombosis, retained blood). Additionally, these CHMs possess antiinflammatory, immune response inhibition, anti-platelet aggregation, and antiperoxidative properties, as well as functions that inhibit proliferation and migration of vascular smooth muscle cells [18–21]. Reviews on the efficacy of CHMs in preventing restenosis after PCI have been published in Chinese [22–24]. But the evidence supporting or disproving the benefits of CHMs is not robust because of methodological deficiencies of those reviews. Our study presents a more vigorous attempt to examine the existing studies to draw more useful conclusions about the safety and efficacy of CHMs in preventing restenosis post-PCI.

## 2. Methods

### 2.1. Inclusion Criteria

Only randomized controlled trials (RCTs) were included regardless of being published or unpublished. We focused on trials with participants diagnosed with major angiographic criteria-documented [25] coronary artery disease who were eligible for PCI regardless of gender, age, and ethnic origin.

The treatment group in the trials was treated with any CHM, or CHM plus RWM with at least 1 month of therapy regardless of dosage. The control group was treated with the same RWM based on the Chinese Society of Cardiology guidelines for percutaneous transluminal coronary intervention [25], or the same RWM plus placebo. “Chinese herbal medicines” include extracts from mixtures of herbs, single herbs, Chinese prepared medicines, or a compound of herbs that is prescribed by a Chinese medicine practitioner [26].

Primary outcome measures were restenosis, cardiac death, and adverse events occurring in at least 3 months of followup. Secondary outcome measures included recurrent angina, acute myocardial infarction (AMI), revascularization, repeat PCI, coronary artery bypass graft (CABG), minimal luminal diameter (MLD), late loss of lumen (LLL), net gain in lumen diameter (NG), and lesion area net gain (LANG) after PCI, and quality of life during at least 1 month of followup.

### 2.2. Study Identification and Assessment in Included Studies

Two authors (G.-H. Zheng, H.-Y. Chen) independently identified studies through searches of the Cochrane Library (Issue 4, 2010), PubMed (January1966 to December 2010), Embase (January 1980 to December 2010), the China Biological Medicine Database (CBM, January 1980 to December 2010), Chinese Scientific Journal Database (VIP, January1989 to December 2010), China National Knowledge Information database (CNKI, January 1994 to December 2010) and Chinese Medical Citation Index (CMCI, January 1999 to December 2010) with free terms related to heart disease and CHM (e.g., “coronary heart disease” OR “coronary artery disease” OR “cardiovascular disease” OR “percutaneous coronary intervention” OR “stent” OR “stenosis” OR “restenosis” OR “CHD” OR “CVD” OR “CAD” OR “MI” “Chinese” OR “herbal” OR for English databases. The Chinese counterpart terms were used for Chinese databases. The reference list of each relevant article was searched for further studies. Unpublished literature was searched using Chinese Master's Theses Full-text Database (CMFD), China Doctor Dissertation Full-text Database (CDFD) and China Proceedings of Conference Full-text Database (CPCD).

Risk of bias in included studies was assessed using The Cochrane Collaboration's tool for assessing risk of bias. Six criteria were applied: adequate sequence generation, concealment of allocation, blinded of primary outcomes, adequately addressed incomplete outcome data, free from selective reporting, and free of other risk of bias [27]. In addition, we assessed the baseline characteristics between the comparison groups.

### 2.3. Data Extraction

Two authors (G.-H. Zheng, J.-F. Chu) independently selected those trials that met the inclusion criteria and extracted details on randomization, allocation concealment, blinding, intent to treat analysis, numbers lost to followup, patient demographics, methods, interventions, outcomes, and results. Missing data were obtained from the original authors when possible.

### 2.4. Data Analysis

Heterogeneity across studies was tested using a standard **χ*^2^* test [28] and Higgins *I* ^2^ [29]. When heterogeneity was not significant (*P* ≥ 0.1), the results were pooled using a fixed effect model and the Mantel-Haenszel test. Otherwise, a random effect model and the Dersimonian and Laird method were applied [30]. The results were reported as risk ratio (RR) with corresponding 95% confidence interval (CI) for dichotomous data. If continuous data were available, weighted mean difference or standardized mean difference was calculated [31]. All data were analyzed using the statistical software RevMan 5.0.1 (Oxford, England) of The Cochrane Collaboration, and all *P* values were two sided.

## 3. Results

### 3.1. Study Identification

**Figure 1:**
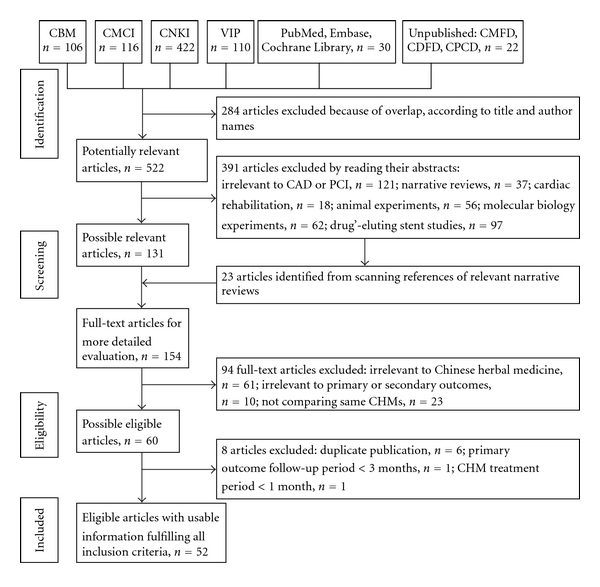
Literature search and selection. Abbreviations: CBM: China Biological Medicine Database; CDFD: China Doctor Dissertation Full-Text Database; CMCI: Chinese Medical Citation Index; CMFD: Chinese Master's Theses Full-Text Databases; CNKI: China National Knowledge Infrastructure; CPCD: China Proceedings of Conference Full-text Database; VIP: VIP Database.

Eligible literature was screened and identified (Figure 1). A total of 806 records were retrieved. Of these, full-text evaluation was conducted on 154 studies. This was followed by elimination of 102 studies: irrelevant to CHM (*n* = 61); irrelevant to the primary or secondary outcomes (*n* = 10); control group combined with another CHM (*n* = 23); duplicate publication (*n* = 6) [32–37]; primary outcomes <3 months' followup (*n* = 1)[38]; CHM treatment <1 month treatment (*n* = 1) [39]. Finally, 52 RCTs with a total of 4905 patients in treatment and control groups, fulfilled the inclusion criteria [40–91]. All studies were conducted in China from 1979 to 2010.

### 3.2. Characteristics of Included RCTs

Table 1 summarizes the characteristics of included studies. Average age of patients in the included studies ranged from 51.2 to 72 years. Each trial had more males than females. The diagnostic criteria of CAD were mainly based on coronary angiography criteria. All patients successfully underwent PCI.

Four trials [45, 67, 71, 81] were randomized double blind, and placebo controlled comparing RWM plus CHM *versus* the same RWM plus placebo. The remaining trials were designed comparing CHM plus RWM *versus* the same RWM alone. The dosage and types of RWM were prescribed according to Chinese Society of Cardiology guideline recommendations [25]. In the trials, 34 kinds of CHMs were used, and the period of treatment with a CHM was at least 1 month. Followup after PCI ranged from 3 to 12 months, with 6 months in the majority of studies.

Restenosis was assessed using angiography in 40 trials. Of these, 10 studies reported angiography assessments at the end of at least 3 months after PCI. Adverse events caused by CHMs were reported in 21 studies, but none of the studies underwent statistical analysis. Recurrent angina was reported in 33 studies. Major cardiac events were reported in 16 trials, and 10 trials reported cardiac death after followup of at least 6 months. Only 2 studies assessed quality of life using the Short-Form 36 (SF-36) Health Survey and Seattle Angina Questionnaire (SAQ) at the end of 1 month after PCI.

### 3.3. Methodological Quality of Included RCTs

**Figure 2:**
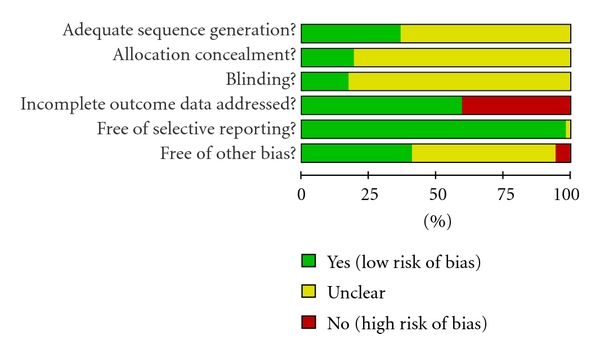
Risk of bias graph depicting proportions of studies with each judgment.

Risk of bias in the studies is shown in Figure 2. Of the 52 studies, 19 studies reported randomization using random number tables or computer random number generator such as SAS. The remaining 33 studies reported “randomly allocating” participants, but the method of randomization was not described. Allocation concealment in 10 of the 52 studies was by sealed, opaque envelopes. In 9 trials, participants and/or outcome assessors were blinded. In most studies, data collection was clearly described and reported, so we judged them as free of selective reporting outcomes. We graded 28 of the 52 studies as “unclear” in terms of free of other bias because there was no evidence of statistical testing of baseline characteristics between comparison groups or the outcome data were incomplete. As a whole, 10 studies [44–46, 53, 67, 71, 75, 76, 81, 85] had low risk of bias with high methodological quality. Most studies were found to be high risk of bias with low methodological quality.

### 3.4. Measures of Effect

#### 3.4.1. Restenosis

In 40 studies on 29 Chinese herbal medicines (CHMs) comprised of 3805 patients, restenosis was assessed using coronary angiography after PCI with at least 3 months' followup (Table 2). Compared to RWM alone, the rate of restenosis was clearly low in patients administered the same RWM plus CHMs. Of 29 CHMs, 9 CHMs (including *Xiong shao capsule, Dan shen, Ge gen shu, Tong mai yu xin pill, Tong xin luo, Wen tong jian, Xue zhi kang, Yi xin capsule, *and *self-prepared guan tong decoction*) showed significant ability in reducing restenosis. In particular, two studies [67, 81] on *Xiongshao *capsule (438 patients) appeared to have reliable results because their designs were randomized, double blinded, and placebo controlled. The overall risk ratio (RR) of restenosis was 0.46 with 95%CI = 0.27 to 0.76 compared to placebo.

#### 3.4.2. Cardiac Mortality

In 10 studies on 9 CHMs, comprised of 628 patients in the treatment group and 667 patients in the control group, cardiac death was included as a measure of effect at the end of 6 months of follow-up after PCI. A pooled analysis of CHM plus RWM versus the same RWM found a statistically significant decrease in risk of cardiac death associated with CHM, though we did not find a significant difference upon subgroup analysis based on different CHMs (Table 3).

#### 3.4.3. Adverse Effects

Adverse effects due to CHMs were not mentioned in 30 studies. In 14 studies, the authors reported that there were no noteworthy adverse events. The remaining 8 studies on 7 CHMs reported adverse events, including gastrointestinal upset, granulocytopenia, elevated alanine transaminase (ALT), aphthous stomatitis, skin pruritus, papular urticaria. Most adverse events were not severe and disappeared without special treatment. With the CHM *Lei gong teng, *adverse events in the treatment group were higher than those in the control group (RR = 37.02, 95%CI = 2.29 to 597.88). With CHM *Si ni tang,* adverse events in the treatment group were lower than that of the control group (RR = 0.38, 95%CI = 0.21 to 0.69). There was no statistically significant difference between the two groups in the remaining 5 CHMs. Pooled analysis could not be done in 8 studies on 7 CHMs because of heterogeneity among the studies.

#### 3.4.4. Recurrent Angina

Recurrent angina 6 months post-PCI was reported as a measure of effect in 33 studies comprised of 3375 patients (Table 4). The rate of recurrent angina in the RWM plus CHM group was lower than that of the same RWM alone group for the CHMs *Dan hong tong mai capsule, Dan shen, Guan tong formula, Lei gong teng, Tong mai yi xin pill, Tong xin luo, Wen tong jian, Xiong shao capsule, Xue fu zhu yu pill, Xue zhi kang, Wen yang huo xue formula*. A statistically significant difference was not observed for each of the remaining CHMs. Pooled results of these trials showed recurrent angina was significantly decreased. For *Xiong shao capsule, *risk of recurrent angina was clearly decreased in the CHM plus RWM group than in the same RWM plus placebo control group.

#### 3.4.5. Major Adverse Cardiac Effects

**Acute Myocardial Infarction (AMI)**

In 15 studies on 9 CHMs, involving 1551 patients, acute myocardial infarction was reported as a measure of effect 6 months after PCI (Table 5). No statistically significant difference was found in each study, but meta-analysis did show decreasing incidence of AMI in the CHM plus RWM group (RR = 0.22, 95%CI = 0.1 to 0.49).

**Revascularization**

Revascularization after the index PCI was reported as a measure of effect in 6 studies on 4 CHMs (*Shu xin yin, Bu xin yin, Yi xin capsule*,* and Xiong shao capsule*) comprised of 716 patients (Table 5). Numbers of revascularization in the CHM plus RWM group was not significantly lower than that in RWM alone group (RR = 0.64, 95% CI = 0.38 to 1.08). However, compared to RWM plus placebo, administering CHM *Xiong shao capsule* plus the same RWM (2 studies with 426 patients) clearly reduced the numbers of revascularization (RR = 0.48, 95%CI = 0.30 to 0.78).

**Repeat PCI**

Repeat PCI was reported as a measure of effect in 9 studies, which involved 8 CHMs with 807 patients (Table 5). Numbers of repeat PCI in the CHM plus RWM group was lower than that of the RWM alone group for *Fu fang dan shen pill* (RR = 0.14, 95%CI = 0.03 to 0.58) and *Tong xin luo* (RR = 0.16, 95%CI = 0.04 to 0.68). The remaining CHMs showed no statistically significant difference between the CHM plus RWM group and RWM alone group. But the overall result of their meta-analyses showed an obvious statistical difference between CHMs plus RWM *versus* RWM groups (RR = 0.37, 95%CI = 0.23 to 0.59).

**Coronary Artery Bypass Graft**

Coronary artery bypass graft (CABG) after index PCI was reported as a measure in 9 studies on 7 CHMs with 1141 patients (Table 5). CHM plus RWM was found to markedly reduce the risk of CABG over the same RWM alone (RR = 0.29, 95%CI = 0.09 to 0.96). But compared to RWM plus placebo, CHM *Xiong shao capsule* plus RWM could not reduce risk of CABG (RR = 0.20, 95%CI = 0.02 to 1.68).

#### 3.4.6. Effect on Angina

Effect on angina was reported as a measure in 6 studies with 522 patients (Table 6). Two studies reported a followup period of 1 month, 2 studies reported a followup of 3 months, and 2 studies reported a followup of 6 months after index PCI. Angina improvement in these studies was defined as “significant improvement,” “improvement,” and “no improvement” based on Chinese herbal medicine clinical research guidelines [92]. To permit overall analysis, we converted these outcomes into dichotomous data. We grouped together “significant improvement” and “improvement” as “effective,” and “no improvement” as “ineffective.” There was no statistically significant difference between CHM plus RWM *versus* RWM groups for every CHM except for *Shen mai gua lou shi xiao powder* (RR = 1.55, 95%CI = 1.11 to 2.17). Furthermore, the results were unsuitable for meta-analysis pooling due to heterogeneity (*I* ^2^ = 60%).

#### 3.4.7. Angiographic Measurements

Follow-up angiography was done on diffuse ISR 6 months after index PCI in 10 studies on 8 CHMs with 811 patients. Baseline information shows that the mean minimal luminal diameter (MLD) before and immediately after index PCI and gain in luminal diameter following stent placement were comparable between the comparison groups.

**Minimum Lumen Diameter (MLD)**

Minimum lumen diameter is defined as smallest diameter of the lesion area being treated [93]. MLD was measured in 7 CHM studies (Table 7). The CHM plus RWM groups showed significant MLD improvement over the same RWM alone or plus placebo for* Fu fang dan shen capsule *(MD (mean difference) = 0.05 mm, 95%CI = 0.04 to 0.26 mm)*, Guan tong formula* (MD = 0.21 mm, 95%CI = 0.06 to 0.36 mm) and *Xiong shao capsule* (placebo control; MD = 0.49 mm, 95%CI = 0.12 to 0.86 mm). No significant difference was evident in the studies on *Lei gong teng, Tong xin fang, Si ni tang*. But in pooling the results of those trials, there was a statistically significant difference (MD = 0.15 mm, 95%CI = 0.05 to 0.24 mm), with patients receiving CHMs more likely to have increased MLD than those in the control group.

**Late Loss of Lumen (LLL)**

Late loss of lumen is defined as the decreased amount in lumen diameter after PCI, which is calculated by subtracting MLD at followup from MLD immediately post-procedure [93]. LLL was measured in 6 studies on 5 CHMs after 6 months' followup (Table 7). Five studies on 4 CHMs (*Guan tong formula, Tong xin luo, Shu xue tong injection, *and* Lei gong teng*) reported significantly better results for reducing late loss of lumen in the treatment group over the control group. There was no significant difference in the study on *Fu fang dan shen capsule.* The pooled results showed significant difference (MD = −0.24 mm, 95%CI = −0.34 to −0.15 mm).

**Net Gain in Lumen Diameter (NG)**

Net gain in lumen diameter is defined as the net increase in MLD after PCI [93]. NG after 6 months' followup was measured in 4 studies on 4 CHMs (Table 7). Significant difference was found between the treatment group plus RWM and the control group with the same RWM alone for *Guan tong formula *(MD = 0.21 mm, 95%CI = 0.08 to 0.34 mm) and *Shu xue tong* injection (MD = 0.25 mm, 95%CI = 0.09 to 0.41 mm). The pooled results of meta-analyses, suggested that CHMs plus RWM increased net gain of lumen compared to using RWM alone (MD = 0.2 mm, 95%CI = 0.11 to 0.29 mm). These results are displayed in Table 7.

**Lesion Area Net Gain (LANG)**

Lesion area net gain is defined as the net increase in lumen area before PCI and at followup after PCI [53, 55, 64, 82, 86]. Five studies on 4 CHMs measured LANG at 6 months' followup after PCI (Table 7).The CHMs *Guan tong formula, Tong xin luo, *and* Lei gong teng *demonstrated a significant improvement in LANG. The same result was not found with the fourth CHM,* Fu fang dan shen* capsule. The pooled results of meta-analysis were statistically significant (MD = 0.69 mm^2^, 95%CI = 0.52 to 0.87 mm^2^).

#### 3.4.8. Quality of Life

Two studies [45, 71] with randomized, double-blind and placebo-control design reported quality of life 1 month after PCI using the Chinese versions of the Seattle Angina Questionnaire (SAQ) and Short-Form 36 (SF-36) Health Survey. The SAQ is a 19-item self-administered questionnaire that assesses how patients with CHD are fairing in five dimensions: physical limitation (PL), angina stability (AS), angina frequency (AF), treatment satisfaction (TS), and disease perception (DP). The results of comparing *Xue fu zhu yu capsule* plus RWM with the same RWM plus placebo suggested that receiving CHM had a positive effect on AS (MD = 12.13,95%CI = 4.61 to 19.65) and TS (MD = 12.13, 95%CI = 6.19 to 17.87). However, in the study with* Tong guan capsule*, the results were contrary to that of *Xue fu zhu yu capsule:* AS (MD = −36.2, 95%CI = −48.97 to −23.43), PL (MD = −11.32, 95%CI = −19.38 to −3.26), AF (MD = −35.68, 95%CI = −50.16 to −20.6), TS (MD = −25.03, 95%CI = −33.93 to −16.13), and DP (MD = −9.79, 95%CI = −35.95 to −16.38). Pooled results were not available because of heterogeneity and the *I* ^2^(%) ranging from 88% to 98%.

SF-36 is a survey of patient health comprised of eight multiple-item scales measuring these dimensions: physical function (PF), role-physical (RP), bodily pain (BP), general health (GH), vitality (VT), social function (SF), role-emotional (RE), and mental health (MH). There is also a single-item measure that assesses health transition (HT). Patients receiving *Tong guan capsule* had low scores on all dimensions except for RP when compared to placebo control. Patients administered *Xue fu zhu yu capsule* scored high only on the RE dimension when compared to placebo control. We did not find any statistically significant difference between the treatment and placebo groups in the remaining dimensions. As with the SAQ, pooled results were not available because of heterogeneity with *I* ^2^(%) ranging from 85% to 98%.

## 4. Discussion

In this systematic review, 52 studies accounting for 4905 CAD patients who underwent PCI were identified. Definitive randomization was found in 19 studies and 33 studies were found to be lacking definitive randomization. For the latter, we attempted to contact authors by telephone or e-mail for further information. But most replies were unsatisfactory and did not resolve our questions; other authors did not reply. Therefore, as a whole, the included studies were of low quality. Of all studies, only four were designed to compare CHMs plus RWM versus the same RWM plus placebo [45, 67, 71, 81]. The remaining studies were designed to compare CHMs plus RWM versus the same RWM alone.

In the primary outcomes, 40 studies with 3805 patients assessed in-stent restenosis after PCI. Twenty of these studies involving 10 CHMs showed clear evidence of decrease in restenosis rate. Furthermore, 13 of these studies were of low or moderate risk of bias over a minimum 6 months' followup (*Xiong shao capsule *[67, 80, 81]*, Dan shen *[44, 52, 85]*, Guan tong formula *[54]*, Bu xin yin *[76]*, Jiang lian he ji *[63] *Shu xin yin *[75]*, Tong guan capsule *[70]*, self-prepared guan tong decoction *[83], and *Xue yu tong he ji *[69],). Therefore, a moderate definitive conclusion can be drawn that CHMs are beneficial for preventing coronary restenosis after PCI. In particular, the CHM *Xiong shao capsule*, which was studied in 4 trials with 613 patients showed strong evidence with low risk of bias in preventing restenosis [40, 67, 80, 81]. Other CHMs including *Dan shen *[44, 50, 52, 85, 86] and *Tong xin luo capsule *[55, 79, 82, 90] showed the same significant result. But a definitive conclusion cannot be drawn because of limited numbers of patients in these trials as well as their low methodological quality.

 We were unable to conclude whether CHMs decrease cardiac mortality during 6 months' followup after PCI. Although the result of meta-analysis showed a statistically significant difference between comparison groups, we could not assess a similar result in the analysis of any single relevant trial. This may be due to the limited patient numbers in these studies.

Adverse events in the treatment groups were generally higher than in controls for 5 CHMs, but a statistically significant difference was found in only one study on the CHM *Lei gong teng *[43]. Adverse reactions in these studies were reported as mild. Therefore, we feel further investigation is needed to confirm these reports.

In this review we also examined secondary outcomes, measures of effect post-PCI. Recurrent angina was followed up for 6 months in 33 studies involving 22 CHMs. In 21 of these studies with 11 CHMs, patients in the treatment group had significantly lower incidence of recurrent angina than those in the control group. *Dan shen* (capsule or pill) in 5 studies [44, 50, 57, 85, 86], *Xiong shao capsule* in 4 studies [40, 67, 80, 81], *Tong xin luo capsule* in 3 studies [79, 82, 90], and *Tong mai yu xin concentrated pill *in 2 studies [60, 62] were significantly better than the control at reducing recurrent angina after PCI. Minimum lumen diameter in 7 studies with 6 CHMs, late loss of lumen in 6 studies with 5 CHMs, net gain in lumen diameter in 4 studies with 4 CHMs, and lesion area net gain in 5 studies with 4 CHMs were measured, with results of meta-analyses being statistically significant. Studies on the following CHMs revealed angiographic results in the treatment groups were significantly better than in the control group: *Guan tong formula *[54] for minimum lumen diameter, late loss of lumen, net gain in lumen diameter, and lesion area net gain; *Tong xin luo capsule *[82] for late loss of lumen and area net gain in lumen diameter; *Shu xue tong *[56] for late loss of lumen and area net gain in lumen diameter; *Xiong shao capsule *[67] for minimum lumen diameter; *Lei gong teng *[64] for late loss of lumenand lesion area net gain.

Our meta-analysis found possible benefit in Chinese herbal medicine compared to control in the rates of restenosis, cardiac mortality, recurrent angina, and in MLD, NG, LLL, and LANG. Specifically, the CHMs *Xiong shao capsule *and *Dan shen *appeared to markedly reduce rates of restenosis and recurrent angina, and the CHM *Tong xin luo* was found to significantly reduce restenosis, recurrent angina, LLL, and LANG. The baseline characteristics of most studies, such as age, gender, severity of CAD, degree of coronary stenosis before PCI, and stent type were not significantly different between the treatment and control groups. Nevertheless, concluding that CHMs have definitive preventive effects on restenosis after PCI would be premature because most of the studies were of low quality with shortcomings such as inadequate concealment, nonreporting of dropouts, and their incomplete outcomes data point to the possibility of bias. Additionally, clinical heterogeneity was apparent because different categories of CHMs were used.

The key limitations of our review were quality of the included studies. Ideally, RCTs should adhere to known research design standards. For example, the medication, dosage, and course should be identical in the control groups, and when including patients with different levels of illness, the trial should use stratified randomization. Our examination of these studies did not find enough details of these characteristics though most of the included studies did report comparable baselines between comparison groups. Details about randomization methodology were also lacking. In the 52 studies we reviewed, 19 trials reported randomization using a random number table or computer random number generator such as SAS software, and 10 trials mentioned using sealed, opaque envelope concealment without further explanation. In addition, in some studies post-PCI angiographic assessment for restenosis was not carried out in comparable patient numbers between the study groups. For example, the authors of one study reported 30 patients in the treatment group and 45 patients in the control group. Post-PCI angiography was done on only 19 patients with 24 lesion vessels in the treatment group and on 26 patients with 29 lesion vessels in the control group, with 8 vessels in the treatment group and 13 vessels in control group determined to have restenosis [64]. No explanation was given as to why angiographic assessment was not carried out on comparable patient numbers. Thus this type of incomplete outcome data can lead to selection bias.

Studies that involve therapeutic trials should also report adverse events regardless of whether or not they occurred. Reporting of adverse effects is very important for evaluating the safety of interventional measures even though there is no certainty that the adverse event is related to the interventional measure. Furthermore, adverse events can affect study dropout rates. In our review, only 22 of the 52 trials we investigated reported adverse events, rendering it difficult to systematically evaluate the safety of CHMs for restenosis.

Most of the 52 studies did not mention type of stent deployed, bare metal versus drug eluting. Therefore, the level of effectiveness of CHMs is unknown when different stent types are used. Future research on this topic will help elucidate this.

Another area that did not receive attention in the studies is the effect of diabetes on restenosis. Persons with diabetes who undergo PCI with stent placement have a high rate of restenosis [94, 95]. Though diabetes was included in baseline patient characteristics in the treatment and control groups in most of the 52 studies of this review, none of the reports indicated the impact diabetes may or may not have had on restenosis. Furthermore, in China, CHMs are widely used in the treatment of diabetes [26]. Researchers may want to factor in these issues when designing future studies.

## 5. Conclusion and Recommendations

From this review, we conclude CHMs may have moderate efficacy in preventing restenosis following percutaneous coronary intervention with stent placement. This is despite the fact that our investigation revealed unclear methodological quality, clinical heterogeneity, and some possible bias in the identified studies. Among the CHMs, *Xiong shao capsule* appears to be somewhat effective in preventing restenosis because studies involving this CHM were of low bias and had sufficient patient numbers. The CHM* Dan shen (capsule or pill)* appears to have latent beneficial efficacy in preventing restenosis because there were relatively more studies and patient numbers for this CHM. Therefore, we recommend that *Dan shen *should be a priority for further research. We did not find evidence of a beneficial effect for administering CHMs to prevent major adverse cardiac effects due to restenosis after PCI.

Future trials on CHMs as therapy to prevent restenosis post-PCI need to adhere to established design standards to overcome the limitations presented in this review. In particular, they should ensure adequate concealment of allocation and blinding of primary outcomes assessors.

## Figures and Tables

**Table 1:** Characteristics of baseline, diagnosis, intervention, and outcomes in included studies.

Author, year	No. of patients (T/C)	Baseline characteristics of treatment and control groups	Intervention		CAD diagnostic guidelines	PCI type	Treatment course (mos)	Followup (mos)	Outcomes
			Control	Treatment					
An et al. 2009 [41]	100 (60/40)	Similar in age and sex between the two groups (narrative only)	RWM	RWM plus Yi qi hua yu fang	AG	Stent	3	3	Restenosis
Bao et al. 2005 [42]	76 (32/44)	Age, sex, hypertension, diabetes, hyperlipidemia, smoking, lesion distribution, stent number	RWM	RWM plus Ge gen shu tablet	AG	Stent	3	6	Restenosis
Chen et al. 2005 [43]	80 (40/40)	Age, sex, hypertension, diabetes, hyperlipidemia, smoking, lesion number	RWM	RWM plus Lei gong teng glycosides	AG	Stent	6	6	Restenosis, repeat intervention, MLD
Chen et al. 2005 [44]	86 (43/43)	Age, sex, CAD classification, results and characteristics of AG before PCI	RWM	RWM plus Dan shen injection	AG	Stent	1	6	Restenosis, death, recurrent angina, AMI
Chu et al. 2009 [45]	57 (28/29)	Age, sex, hypertension, diabetes, smoking, course of CAD, BMI, blockage number	RWM plus placebo	RWM plus Xue fu zhu yu capsule	AG	Stent	1	1	Quality of life
Cui et al. 2010 [46]	97 (51/46)	Age, sex, CAD classification, comorbidities, lesion number, stent number	RWM	RWM plus Liang xue shen ji fang	AG	Stent	2	6	Restenosis, death, recurrent angina, CABG, repeat PCI
Dan 2009 [47]	120 (60/60)	Age, sex, hypertension, diabetes, CAD classification, stent type	RWM	RWM plus Wen yang huo xue fang	AG	PTCA or stent	3	12	Recurrent angina and accompanying symptoms
Ding et al. 2007 [48]	55 (26/29)	Average age, sex, lesions, stent (narrative only)	RWM	RWM plus Xue zhi kang capsule	AG	Stent	6	6	Restenosis
Feng et al. 2006 [49]	61 (30/31)	Similar in baseline information between the two groups (narrative only)	RWM	RWM plus Tong xin fang	AG	PTCA	6	6	Restenosis, MLD
Fu 2009 [50]	80 (40/40)	Lesion characteristics and degree, stent type (narrative only)	RWM	RWM plus Fu fang dan shen pill	AG	Stent	6	6	Restenosis, recurrent angina, AMI, repeat intervention, CABG
Gu et al. 2007 [51]	60 (30/30)	Age, sex, complications, lesion number and type, stent type	RWM	RWM plus Xue fu zhu yu pill	AG	Stent	6	6	Restenosis, recurrent angina
Guo et al. 2009 [52]	80 (40/40)	Age, sex, complications, lesion number	RWM	RWM plus Dan shen tablet	AG	DES	6	6	Restenosis, death, CABG, repeat PCI
Han 2008 [53]	60 (30/30)	Age, sex, patient characteristics, hypertension, diabetes, smoking, lesion characteristics	RWM	RWM plus Xue sai tong capsule	AG	Stent	3	3	Recurrent angina
He et al. 2010 [54]	120 (60/60)	Age, sex, hypertension, diabetes, course of CAD, lesion characteristics	RWM	RWM plus Guan tong fang	AG	BMS	5	6	Restenosis, recurrent angina, MLD, LLS, NG, LANG
Kai et al. 2008 [55]	72 (36/36)	Age, sex, CAD characteristics, lesion characteristics (narrative only)	RWM	RWM plus Tong xin luo capsule	AG	Stent	6	6	Restenosis, LLS, NG, LANG
Li et al. 2004 [56]	57 (37/20)	Age, sex, hyperlipidemia, diabetes, smoking, lesion distribution and characteristics	RWM	RWM plus Shu xue tong injection	AG	Stent	1	6	Recurrent angina, MLD, LLS, NG
Li et al. 2010 [57]	80 (42/38)	Age, sex, lesion characteristics and degree, stent type (narrative only)	RWM	RWM plus Fu fang dan shen pill	AG	Stent	6	6	Recurrent angina, MI, repeat PCI, CABG
Li et al. 2005 [58]	52 (26/26)	Age, sex, hypertension, diabetes, smoking, lesion number	RWM	RWM plus Tong guan capsule	AG	PTCA or stent	3	6	Clinical effect of AP
Li et al. 2006 [59]	80 (40/40)	Age, hypertension, diabetes, lesion number and site	RWM	RWM plus Shi ni decoction	AG	Stent	6	6	Restenosis, repeat intervention, MLD
Li et al. 2008 [60]	121 (61/60)	Age, sex, hypertension, diabetes, smoking, lesion number and lsite	RWM	RWM plus Tong mai yi xin pill	AG	PTCA	6	6	Restenosis, recurrent angina
Li et al. 2004 [61]	70 (36/34)	Age, sex, complications, lesion number (narrative only)	RWM	RWM plus Xing mai fu tong decoction	Meeting guide	Stent	3	6	Restenosis
Li 2009 [62]	151 (76/75)	Age, sex, hypertension, diabetes, smoking, lesion characteristics	RWM	RWM plus Tong mai yi xin pill	AG	Stent	6	6	Restenosis, recurrent angina
Li and Niu 2008 [63]	59 (36/33)	Age, sex, hypertension, diabetes, smoking, lesion number and degree of blockage	RWM	RWM plus Jiang lian he ji	AG	PTCA	3	6	Restenosis, recurrent angina
Liu et al.2002 [64]	75 (30/45)	Age, sex, hypertension, diabetes, smoking, lesion number and degree of blockage	RWM	RWM plus Lei gong teng glycosides	AG	Stent	6	6	Restenosis, recurrent angina, MACE, MLD, LLS, NG, LANG
Liu et al. 2004 [65]	165 (63/102)	Age, sex, hyperlipidemia, diabetes,, lesion number and characteristics	RWM	RWM plus Chuan qiong qing	AG	PTCA or stent	6	6	Restenosis, death
Liu et al. 2007 [66]	60 (30/30)	Sex, hypertension, diabetes	RWM	RWM plus Yi qi huo xue fang	AG	PTCA	6	6	Recurrent angina
Lu et al. 2006 [67]	124 (62/62)	Age, sex, hypertension, diabetes, hyperlipidemia, lesion number, location and degree of stenosis, stent number	RWM	RWM plus Xiong shao capsule	AG	Stent	6	6	Restenosis, recurrent angina, AMI, CABG, MLD
Ma et al. 2009 [68]	92 (50/42)	Age, sex, complications, lesion number (narrative only)	RWM	RWM plus Guan mai zai tong	Meeting guide	Stent	3	3	Restenosis
Niu et al. 2003 [69]	36 (18/18)	Baseline patient characteristics (narrative only), hypertension, diabetes, smoking, characteristics and degree of stenosis	RWM	RWM plus Xue yu tong he ji	AG	PTCA or stent	6	6	Restenosis, recurrent angina
Qi et al. 2003 [70]	50 (30/20)	Baseline patient characteristics, lesion number and site (narrative only)	RWM	RWM plus Tong guan capsule	AG	PTCA	6	6	Restenosis, recurrent angina
Qiao et al. 2006 [71]	59 (30/29)	Baseline patient characteristics, hypertension, diabetes, smoking, lesion number and degree of stenosis	RWM plus placebo	RWM plus Tong guan capsule	AG	PTCA	1	1	Quality of life
Shi et al. 1997 [72]	73 (35/38)	Age, sex, hypertension, diabetes, lesion characteristics	RWM	RWM plus Xue fu zhu yu pill	AG	PTCA	6	6	Restenosis, recurrent angina
Wang et al. 2009 [73]	101 (54/47)	Age, sex, course of CAD, degree of blockage, complications	RWM	RWM plus Yi qi huo xue formula	AG	PTCA or stent	3	3	Angina symptoms
Wang et al. 2006 [74]	92 (62/30)	Age, sex, course of CAD (narrative only)	RWM	RWM plus Shan shen tong mai ji	AG	PTCA or stent	1	1	Angina symptoms
Wang et al. 2002 [75]	44 (20/24)	Baseline patient characteristics, CAD course, AG characteristics (narrative only)	RWM	RWM plus Shu xin yin	AG	Stent	6	6	Restenosis, recurrent angina, MI, MACE
Wang et al. 2003 [76]	44 (20/24)	Age, sex, hypertension, diabetes, smoking, AG characteristics	RWM	RWM plus Bu xin yin	AG	Stent	6	6	Restenosis, recurrent angina, AMI, revascularization
Wang and Gao 2004 [77]	94 (41/53)	Age, sex, hypertension, diabetes, smoking, lesion number and type	RWM	RWM plus Yi xin capsule	AG	Stent	6	6	Restenosis, recurrent angina, AMI, revascularization,
Wang et al. 2010 [78]	40 (20/20)	Baseline characteristics similar (narrative only)	RWM	RWM plus Ku lie zhi injection	AG	Stent	6	6	Restenosis, death, AMI
Xiao et al. 2007 [79]	132 (62/70)	Age, sex, hypertension, diabetes, arrhythmia, stent number	RWM	RWM plus Tong xin luo	AG	BMS	6	6	Restenosis, recurrent angina, death, AMI, MACE
Xu et al. 2000 [80]	65 (28/37)	Age, sex, hypertension, diabetes, lesion number and site	RWM	RWM plus Xiong shao capsule	AG	PTCA or stent	6	6	Restenosis, recurrent angina, AMI
Xu et al. 2002 [40]	108 (53/55)	Age, sex, CAD classification	RWM	RWM plus Xiong shao capsule	AG	PTCA or stent	6	6	Restenosis, recurrent angina, AMI, CABG, revascularization
Chen et al. 2006 [81]	314 (157/157)	Age, sex, hypertension, diabetes, lesion number and site	RWM plus placebo	RWM plus Xiong shao capsule	AG	PTCA or stent	6	6	Restenosis, recurrent angina, death, CABG
Yao et al. 2006 [82]	76 (38/38)	Age, sex, lesion number and site	RWM	RWM plus Tong xin luo	AG	PTCA	6	6	Restenosis, recurrent angina, LLS, NG, LANG
Yu et al. 1998 [84]	84 (43/41)	Baseline patient characteristics, hypertension, diabetes, smoking, lesion number and degree of blockage	RWM	RWM plus Xue fu zhu yu pill	AG	Stent	6	6	Restenosis, recurrent angina and accompanying symptoms
Z. A. Yu and S. Y. Yu2006 [85]	82 (42/40)	Age, sex, course of CAD, lesion number and site and degree of blockage	RWM	RWM plus Dan shen tablets	AG	Stent	6	6	Restenosis, recurrent angina, AMI, death
Yi et al. 2005 [83]	40 (20/20)	Age, sex, lesion numbers and sites, history of hypertension and diabetes	RWM	RWM plus Guan tong jian ji	AG	PTCA or stent	6	6	Restenosis, recurrent angina
Zhang et al. 2007 [88]	63 (33/30)	Age, sex, hypertension, diabetes, smoking, lesion site, angina,	RWM	RWM plus Shen mai gua lu shi xiao san	AG	Stent	1	4	Angina symptoms
Zhang et al. 2006 [87]	500 (250/250)	Age, sex, hypertension, diabetes, hyperlipidemia, BMI, degree of blockage (narrative only)	RWM	RWM plus Wen tong jian	AG	PTCA or stent	6	6	Restenosis, recurrent angina
Zhao et al. 2009 [89]	68 (35/33)	Age, sex, hypertension, diabetes, stenosis and lesion diameter of vascular lesion	RWM	RWM plus Shen qi tang	AG	Stent	6	6	Restenosis
Zheng et al. 2004 [86]	66 (36/30)	Baseline patient characteristics, lesion number and characteristics (narrative only)	RWM	RWM plus Fu fang dan shen pill	AG	Stent	6	6	Restenosis, recurrent angina, LLS, NG, LANG
Zhou and Guo 2007 [90]	136 (70/66)	Baseline patient characteristics, lesion characteristics and degree of blockage, stent type (narrative only)	RWM	RWM plus Tong xin luo	AG	Stent	6	6	Restenosis, recurrent angina, AMI, CABG, repeat PCI
Zhu et al. 2009 [91]	138 (70/68)	Age, sex, hypertension, diabetes, hyperlidemia, smoking, lesion number and degree of blockage, stent number	RWM	RWM plus Dan hong tong mai capsule	AG	DES	6	6	Restenosis, recurrent angina, death, AMI, MACE, CABG, repeat PCI

AG = angiography; BMI = body mass index; BMS = bare metal stent; CABG = coronary artery bypass graft; CAD = coronary artery disease; DES = drug eluting stent; LANG = lesion area net gain; LLL = late loss of lumen; MLD = minimum lumen diameter; NG = net gain in lumen diameter; PCI = percutaneous coronary intervention; PTCA = percutaneous transluminal coronary angioplasty; RWM = routine Western medicine; T/C = treatment/control; TCM = traditional Chinese medicine.

**Table 2:** Risk ratios of Chinese herbal medicines administered to prevent restenosis after PCI.

Chinese herbal medicine	Number of studies	Number of patients	Risk ratio (RR)	95% CI of RR
Bu xin yin plus RWM *versus* RWM	1 [76]	44	0.69	0.23~2.01
Chuan qiong qing plus RWM *versus* RWM	1 [65]	165	0.51	0.19~1.31
Dan hong tong mai capsule plus RWM *versus* RWM	1 [91]	103	0.35	0.10~1.23
Dan shen plus RWM *versus* RWM	5 [44, 50, 52, 85, 86]	388	0.27	0.14~0.52
Ge gen shu plus RWM *versus* RWM	1 [42]	76	0.23	0.06~0.95
Guan mai zhai tong decoction plus RWM *versus* RWM	1 [68]	92	0.32	0.09~1.11
Guan tong formula plus RWM *versus* RWM	1 [54]	120	0.43	0.18~1.04
Jia wei xue fu zhu yu particle plus RWM *versus* RWM	1 [51]	40	0.75	0.27~2.07
Jiang lian he ji plus RWM *versus* RWM	1 [63]	26	0.57	0.11~2.87
Ku lie zhi injection plus RWM *versus* RWM	1 [78]	40	1.00	0.07~14.9
Lei gong teng plus RWM *versus* RWM	2 [43, 64]	133	0.75	0.40~1.40
Liang xue shen ji formula plus RWM *versus* RWM	1 [46]	97	0.45	0.12~1.70
Shen qi decoction plus RWM *versus* RWM	1 [89]	68	0.47	0.09~2.40
Shu xin yin plus RWM *versus* RWM	1 [75]	44	0.69	0.23~2.01
Si ni tang plus RWM *versus* RWM	1 [59]	63	0.55	0.14~2.09
Tong guan capsule plus RWM *versus* RWM	1 [70]	25	0.67	0.26~1.72
Tong mai yu xin plus RWM *versus* RWM	2 [60, 62]	272	0.39	0.21~0.70
Tong xin fang plus RWM *versus* RWM	1 [49]	61	1.03	0.23~4.72
Tong xin luo plus RWM *versus* RWM	4 [39, 79, 82, 90]	328	0.34	0.20~0.59
Wen tong jian plus RWM *versus* RWM	1 [87]	467	0.03	0.00~0.49
Xue fu zhu yu pin plus RWM *versus* RWM	2 [72, 84]	157	0.61	0.32~1.16
Xue yu tong he ji plus RWM *versus* RWM	1 [69]	36	1.0	0.16~6.35
Xue zhi kang plus RWM *versus* RWM	1 [48]	55	0.30	0.09~0.99
Xing mai fu tong decoction plus RWM *versus* RWM	1 [61]	70	0.71	0.17~2.94
Xiong shao capsule plus RWM *versus* RWM	2 [40, 80]	173	0.43	0.23~0.81
Yi qi hua yu formula plus RWM *versus* RWM	1 [41]	100	0.13	0.02~1.10
Yi xin capsule plus RWM *versus* RWM	1 [77]	94	0.50	0.27~0.91
Self-prepared guantong decoction plus RWM *versus* RWM	1 [83]	30	0.40	0.16~1.00
*Overall (CHMs plus RWM versus RWM)*	**38**	**3367**	**0.43**	**0.36~0.51**
Xiong shao capsule plus RWM *versus* RWM plus placebo	2 [67, 81]	438	0.46	0.27~0.76
*Overall (CHMs plus RWM versus RWM plus placebo)*	**2 **	**438**	**0.46**	**0.27~0.76**

CHM = Chinese herbal medicine; CI = confidence interval; RWM = routine Western medicine.

**Table 3:** Risk ratios of Chinese herbal medicines administered to prevent cardiac mortality after PCI.

Chinese herbal medicine	Number of studies	Number of patients	Risk ratio (RR)	95% CI of RR
Bu xin yin plus RWM *versus* RWM	1 [76]	44	0.4	0.02~9.24
Chuan qiong qing plus RWM *versus* RWM	1 [65]	165	0.23	0.01~4.38
Dan hong tong mai capsule plus RWM *versus* RWM	1 [91]	138	2.92	0.12~70.35
Dan shen plus RWM *versus* RWM	2 [44, 85]	168	0.33	0.03~3.07
Ku lie zhi injection plus RWM *versus* RWM	1 [78]	40	0.33	0.01~7.72
Liang xue shen ji formula plus RWM *versus* RWM	1 [46]	97	0.15	0.02~1.2
Shu xin yin plus RWM *versus* RWM	1 [75]	44	0.40	0.02~9.24
Tong xin luo plus RWM *versus* RWM	1 [79]	132	0.38	0.02~9.06
Wen tong jian plus RWM *versus* RWM	1 [87]	467	0.07	0.00~1.32
*Overall (CHMs plus RWM versus RWM)*	**10**	**1295**	**0.27**	**0.11~0.63**

CHM = Chinese herbal medicine; CI = confidence interval; RWM = routine Western medicine.

**Table 4:** Risk ratios of Chinese herbal medicines administered to prevent recurrent angina after PCI.

Chinese herbal medicine	Number of studies	Number of patients	Risk ratio (RR)	95% CI of RR
Bu xin yin plus RWM *versus* RWM	1 [76]	44	0.60	0.33~1.10
Dan hong tong mai capsule plus RWM *versus* RWM	1 [91]	138	0.24	0.07~0.82
Dan shen plus RWM *versus* RWM	5 [44, 50, 57, 85, 86]	390	0.24	0.15~0.41
Guan tong formula plus RWM *versus* RWM	1 [54]	120	0.27	0.08~0.93
Jia wei xue fu zhu yu particle plus RWM *versus* RWM	1 [51]	60	0.45	0.18~1.15
Jiang lian he ji plus RWM *versus* RWM	1 [63]	69	0.71	0.30~1.70
Lei gong teng plus RWM *versus* RWM	1 [64]	75	0.39	0.17~0.94
Liang xue shen ji formula plus RWM *versus* RWM	1 [46]	97	0.52	0.16~1.65
Shu xue tong injection plus RWM *versus* RWM	1 [56]	43	0.37	0.11~1.25
Shu xin yin plus RWM *versus* RWM	1 [75]	44	0.60	0.33~1.10
Tong guan capsule plus RWM *versus* RWM	1 [70]	50	0.67	0.22~2.01
Tong mai yi xin plus RWM *versus* RWM	2 [54]	272	0.41	0.26~0.65
Tong xin luo plus RWM *versus* RWM	3 [79, 82, 90]	344	0.27	0.13~0.54
Wen tong jian plus RWM *versus* RWM	1 [87]	467	0.30	0.17~0.52
Wen yang huo xue formula plus RWM *versus* RWM	1 [47]	120	0.53	0.38~0.75
Xue fu zhu yu pin plus RWM *versus* RWM	2 [72, 84]	157	0.44	0.25~0.78
Xue yu tong he ji plus RWM *versus* RWM	1 [69]	36	0.67	0.23~1.97
Xue zhi kang plus RWM *versus* RWM	1 [48]	60	0.38	0.16~0.94
Xiong shao capsule plus RWM *versus* RWM	2 [40, 80]	173	0.48	0.31~0.75
Yi qi hua yu formula plus RWM *versus* RWM	1 [41]	60	0.44	0.15~1.29
Yi xin capsule plus RWM *versus* RWM	1 [77]	94	0.46	0.20~1.05
Self-prepared guan tong decoction plus RWM *versus* RWM	1 [83]	40	0.33	0.11~1.05
*Overall (CHM plus RWM versus RWM)*	**31**	**2953**	**0.40**	**0.35~0.47**
Xiong shao capsule plus RWM *versus* RWM plus placebo	2 [67, 81]	422	0.25	0.17~0.37
*CHM plus RWM versus RWM plus placebo*	**2**	**422**	**0.25**	**0.17~0.37**

CHM = Chinese herbal medicine; CI = confidence interval; RWM = routine Western medicine.

**Table 5:** Risk ratios of Chinese herbal medicines administered to prevent MACE after PCI.

Chinese herbal medicine	Number of studies	Number of patients	Risk ratio (RR)	95% CI of RR
*Acute myocardial infarction*				
Bu xin yin plus RWM *versus* RWM	1 [76]	44	0.24	0.01~4.69
Dan hong tong mai capsule plus RWM *versus* RWM	1 [91]	138	0.14	0.01~2.64
Dan shen plus RWM *versus* RWM	2 [44, 85]	168	0.14	0.02~1.11
Fu fang dan shen pill plus RWM *versus* RWM	2 [50, 57]	156	0.32	0.03~2.99
Ku lie zi injection plus RWM *versus* RWM	1 [78]	40	0.33	0.01~7.72
Shu xin yin plus RWM *versus* RWM	1 [75]	44	0.24	0.01~4.69
Tong xin luo plus RWM *versus* RWM	2 [79, 90]	268	0.25	0.03~2.22
Xiong shao capsule plus RWM *versus* RWM	2 [40, 80]	173	0.14	0.02~1.06
Yi xin capsule plus RWM *versus* RWM	1 [77]	94	0.65	0.06~6.88
*Overall (CHMs plus RWM versus RWM) *	**13**	**1125**	**0.22**	**0.10~0.49**
Xiong shao capsule plus RWM *versus* RWM plus placebo	2 [73]	426	0.59	0.08~4.41
*Overall (CHMs plus RWM versus RWM plus placebo) *	**2 **	**426**	**0.59**	**0.08~4.41**
*Revascularization*				
Shu xin yin plus RWM *versus* RWM	1 [75]	44	0.69	0.23~2.01
Bu xin yin plus RWM *versus* RWM	1 [76]	44	0.69	0.23~2.01
Yi xin capsule plus RWM *versus* RWM	1 [77]	94	0.43	0.02~10.26
Xiong shao capsule plus RWM *versus* RWM	1 [40]	108	0.62	0.30~1.30
*Overall (CHMs plus RWM versus RWM) *	**4**	**290**	**0.64**	**0.38~1.08**
Xiong shao capsule plus RWM *versus* RWM plus placebo	2 [67, 81]	426	0.48	0.30~0.78
*Overall (CHMs plus RWM versus RWM plus placebo) *	**2 **	**426**	**0.48**	**0.30~0.78**
* Repeat PCI*				
Dan hong tong mai capsule plus RWM *versus* RWM	1 [91]	138	0.39	0.08~1.94
Dan shen plus RWM *versus* RWM	1 [52]	80	1.0	0.21~4.66
Fu fang dan shen pill plus RWM *versus* RWM	2 [50, 57]	156	0.14	0.03~0.58
Ku lie zi injection plus RWM *versus* RWM	1 [78]	40	0.33	0.01~7.72
Lei gong teng plus RWM *versus* RWM	1 [43]	80	0.56	0.20~1.51
Liang xue shen ji formula plus RWM *versus* RWM	1 [46]	97	0.54	0.14~2.14
Si ni tang plus RWM *versus* RWM	1 [59]	80	0.56	0.20~1.51
Tong xin luo plus RWM *versus* RWM	1 [90]	136	0.16	0.04~0.68
*Overall (CHMs plus RWM versus RWM) *	**9**	**807**	**0.37**	**0.23~0.59**
*CABG*				
Dan hong tong mai capsule plus RWM *versus* RWM	1 [91]	138	0.14	0.01~2.64
Dan shen plus RWM *versus* RWM	1 [52]	80	Not	estimable
Fu fang dan shen pill plus RWM *versus* RWM	2 [50, 57]	156	0.32	0.03~2.99
Liang xue sheng ji formula plus RWM *versus* RWM	1 [46]	97	0.30	0.01~7.22
Tong xin luo plus RWM *versus* RWM	1 [90]	136	0.19	0.01~3.86
Xiong shao capsule plus RWM *versus* RWM	1 [40]	108	1.04	0.07~16.2
*Overall (CHMs plus RWM versus RWM) *	**7**	**715**	**0.29**	**0.09~0.96**
Xiong shao capsule plus RWM *versus* RWM plus placebo	2 [67, 81]	426	0.20	0.02~1.68
*Overall (CHMs plus RWM versus RWM plus placebo) *	**2 **	**426**	**0.20**	**0.02~1.68**

CABG = coronary artery bypass graft; CHM = Chinese herbal medicine; CI = confidence interval; PCI = percutaneous coronary intervention; RWM = routine Western medicine; MACE = major adverse cardiac event.

**Table 6:** Risk ratios of Chinese herbal medicines administered to prevent angina after PCI.

Chinese herbal medicine	Number of studies	Number of patients	Risk ratio (RR)	95% CI of RR
*Followup 1 month*				
Shan shen tong mai he ji plus RWM *versus* RWM	1 [74]	102	1.18	0.95~1.45
Shen mai gua lou shi xiao power plus RWM *versus* RWM	1 [88]	63	1.55	1.11~2.17
Overall (CHMs plus RWM versus RWM)*	2	165	1.31	1.00~1.72
*Followup 3 months *				
Tong guan capsule plus RWM *versus* RWM	1 [58]	52	1.26	0.98~1.64
Yi qu huo xue formula plus RWM *versus* RWM	1 [73]	101	1.07	0.98~1.16
Overall (CHMs plus RWM versus RWM)*	2	153	1.13	0.93~1.36
*Followup 6 months *				
Xue fu zhu yu pill plus RWM *versus* RWM	1 [84]	84	1.02	0.57~1.84
Wen yang huo xue formula plus RWM *versus* RWM	1 [47]	120	1.05	0.99~1.12
Overall (CHMs plus RWM *versus* RWM)	2	204	1.05	0.99~1.12
Total overall (CHMs plus RWM versus RWM)*	**6**	**522**	**1.13**	**1.02~1.26**

*Random effect model

CHM = Chinese herbal medicine; CI = confidence interval; PCI = percutaneous coronary intervention; RWM = routine Western medicine.

**Table 7:** Effect of Chinese herbal medicines on angiographic characteristics after PCI.

Chinese herbal medicine	Number of studies	Number of patients	Mean difference	95% CI of mean difference
*Minimum lumen diameter (mm)*				
Fu fang dan shen plus RWM *versus* RWM	1 [86]	47	0.05	0.04~0.26
Guan tong formula plus RWM *versus* RWM	1 [54]	120	0.21	0.06~0.36
Lei gong teng plus RWM *versus* RWM	2 [43, 64]	119	0.11	−0.13~0.36
Si ni tang plus RWM *versus* RWM	1 [59]	63	0.26	−0.03~0.55
Tong xin fang plus RWM *versus* RWM	1 [49]	61	−0.10	−0.44~0.24
*Overall (CHMs plus RWM versus RWM) *	**6**	**410**	**0.15**	**0.05~0.24**
Xiong shao capsule plus RWM *versus* RWM plus placebo	1 [67]	97	0.49	0.12~0.86
*CHMs plus RWM versus RWM plus placebo *	**1 **	**97**	**0.49**	**0.12~0.86**
*Late loss of lumen (mm)*				
Fu fang dan shen plus RWM *versus* RWM	1 [86]	47	0.00	−0.22~0.22
Guan tong formula plus RWM *versus* RWM	1 [54]	120	−0.16	−0.28~−0.04
Lei gong ten plus RWM *versus* RWM	1 [64]	53	−0.30	−0.48~−0.12
Shu xue tong injection plus RWM *versus* RWM	1 [56]	64	−0.29	−0.44~−0.14
Tong xin luo plus RWM *versus* RWM	2 [55, 82]	148	−0.33	−0.46~−0.21
Overall (CHMs plus RWM versus RWM)*	**6**	**432**	**−0.24**	**−0.34~−0.15**
*Net gain in lumen diameter (mm)*				
Fu fang dan shen plus RWM *versus* RWM	1 [86]	47	0.04	−0.18~0.26
Guan tong formula plus RWM *versus* RWM	1 [54]	120	0.21	0.08~0.34
Lei gong tang plus RWM *versus* RWM	1 [64]	53	0.28	−0.03~0.59
Shu xue tong injection plus RWM *versus* RWM	1 [56]	64	0.25	0.09~0.41
*Overall (CHMs plus RWM versus RWM) *	**4**	**284**	**0.20**	**0.11~0.29**
*Lesion area net gain (mm^2^)*				
Fu fang dan shen plus RWM *versus* RWM	1 [86]	47	0.06	−0.54~0.66
Guan tong formula plus RWM *versus* RWM	1 [54]	120	0.66	0.25~1.07
Lei gong teng plus RWM *versus* RWM	1 [64]	53	0.66	0.41~0.91
Tong xin luo plus RWM *versus* RWM	2 [55, 82]	148	0.99	0.64~1.33
*Overall (CHMs plus RWM versus RWM) *	**5**	**368**	**0.69**	**0.52~0.87**

*Random effect model

CHM = Chinese herbal medicine; CI = confidence interval; PCI = percutaneous coronary intervention; RWM = routine Western medicine.

## Abbreviations and Acronyms

AMI: Acute myocardial infarction
CABG: Coronary artery bypass graft
CAD: Coronary artery disease
CHM: Chinese herbal medicine
DES: Drug-eluting stent
ISR: In-stent restenosis
LANG: Lesion area net gain
LLL: Late loss of lumen
MI: Myocardial infarction
MLD: Minimum lumen diameter
NG: Net gain in lumen diameter
PCI: Percutaneous coronary intervention
RCT: Randomized controlled trials
RR: Risk ratio
RWM: Routine western medicine.
